# Theoretical Derivation of a Telephone-Based Health Coaching Intervention for Promoting Physical Activity and Healthy Nutrition

**DOI:** 10.3390/ijerph20136271

**Published:** 2023-06-30

**Authors:** Angeli Gawlik, Yeliz Nacak, Jens Kleinert, Uwe Konerding, Frank Vitinius

**Affiliations:** 1 Department of Health & Social Psychology, Institute of Psychology, German Sport University Cologne, 50933 Cologne, Germany; kleinert@dshs-koeln.de; 2 Department of Psychosomatics and Psychotherapy, Faculty of Medicine, University Hospital Cologne, University of Cologne, 50931 Cologne, Germany; yeliz.nacak@uk-koeln.de (Y.N.); frank.vitinius@uk-koeln.de (F.V.); 3 Trimberg Research Academy, University of Bamberg, 96045 Bamberg, Germany; uwe.konerding@uni-bamberg.de; 4 Department of Psychology and Psychotherapy, Witten/Herdecke University, 58455 Witten, Germany; 5 Department of Psychosomatic Medicine, Robert-Bosch-Krankenhaus Stuttgart, 70376 Stuttgart, Germany

**Keywords:** intervention, theoretical derivation, physical activity, healthy nutrition, health coaching, type 2 diabetes mellitus, coronary heart disease

## Abstract

Present research regarding interventions to change behavior suffers from insufficient communication of their theoretical derivation. This insufficient communication is caused by the restrictions imposed by most of the relevant scientific journals. This impedes further intervention development. In this article, a telephone-based health coaching (TBHC) intervention is introduced using a format outside these restrictions. This intervention is seen as a combination of (1) the activities performed with the target persons, i.e., its core, and (2) measures to ensure the quality of the intervention. The theoretical derivation of the core is presented. The core is seen to consist of (1) the style of coach–patient interaction and (2) the contents of this interaction. The style of coach–patient interaction was derived from self-determination theory and was concretized using motivational interviewing techniques. The contents of the coach–patient interaction were derived from the health action process approach and were concretized using behavior-change techniques. The derivation led to (1) a set of 16 coaching tools referring to the different states in which a patient might be and containing state-specific recommendations for performing the coaching session, and (2) guidelines for selecting the appropriate coaching tool for each session. To ensure the quality of the intervention, a coach-training program before and supervision sessions during the TBHC were added.

## 1. Introduction

Both physical activity and healthy nutrition are forms of behavior that have a substantial impact on health. They reduce the risk of diseases such as type 2 diabetes mellitus (T2DM) [1,2], coronary heart disease (CHD) [3,4], dementia [5], and cancer [6]. Moreover, they can support the treatment of chronic diseases. For example, the progression of T2DM and CHD can be reduced by increasing levels of physical activity [7,8] and healthy nutrition [1,2,9]. All in all, physical activity and healthy nutrition increase life expectancy [10] and quality of life [11]. Furthermore, since chronic diseases such as T2DM and CHD cause an extreme medical, social, and economic burden [12], promoting physical activity and healthy nutrition might also be an effective measure to reduce this burden. Consequently, there is an urgent need to integrate measures for promoting physical activity and healthy nutrition into regular care. This need has produced a vast amount of research. By far the largest part of this research is concerned with promoting physical activity in persons with T2DM. A review of the reviews published in 2020 refers to 113 evaluation studies in total [13].

All in all, the research outlined above has provided a great deal of useful knowledge. However, this research still has essential shortcomings. In the evaluation studies that form the core of this research, the interventions are usually not described in detail [14]. This impedes replication and the transfer of intervention research into practice. Moreover, in these evaluation studies, the reasons for designing the interventions in the way that they have been designed are not sufficiently reported. To the extent that these reasons appear in empirical evidence, they are usually given by citing the corresponding literature. Yet, assuming that the interventions are derived from theories, this derivation is hardly ever elaborated. Admittedly, several authors of such evaluation studies report that their interventions are based on theories, but these authors hardly ever elaborate as to how they derived their interventions from these theories [15,16,17,18,19,20]. Consequently, an essential part of the reasons for having developed the intervention in the given way remains hidden; therefore, a public discussion of these reasons is impossible. This substantially impedes the process of developing effective interventions because interventions that address new problems cannot completely be derived from prior empirical evidence. They must be derived from theoretical assumptions regarding the relevant underlying mechanisms.

The shortcomings described above are, to a great extent, enforced by the author guidelines provided by the relevant scientific journals. These guidelines focus on the standard format for reports of empirical research. According to this format, an article must consist of (1) an introduction, (2) a description of the empirical and statistical methods, (3) a report of the empirical results, (4) a discussion of the empirical results, and, sometimes, (5) a conclusion. This format allows neither for a detailed description of an intervention nor for an elaboration of its theoretical derivation. Admittedly, some of the relevant journals allow for theoretical articles. However, according to the corresponding guidelines, these articles must focus on the theories themselves. These guidelines do not allow authors to focus on a specific intervention and to discuss theories only in the context of their relationship to this intervention. In other words, the present guidelines essentially impede the effective further development of interventions. Therefore, improving further intervention development requires taking a step outside these guidelines and attempting different article formats.

The main objective of this article is to attempt a format that allows for a detailed description of an intervention and, especially, its theoretical derivation. An article according to this format consists of four parts: (1) an introduction, (2) a presentation of the theoretical and empirical fundaments of the intervention, (3) a detailed description of the intervention, including its theoretical derivation, and (4) a discussion. The intervention considered here is telephone-based health coaching (TBHC), acting as one component of a more comprehensive intervention addressing persons enrolled in a German disease management program for T2DM and/or for CHD [21]. In addition to the TBHC, the complete intervention program also includes peer-support-group meetings, personalized patient feedback, and access to a web portal [21]. The TBHC itself is only administered to a sub-sample of those persons addressed by the complete intervention. This sub-sample consists of persons with low health competency, as measured using the brief health literacy scale (BHLS) [22], and/or low activation level, as measured using the patient activation measure (PAM) [23,24]. The TBHC aims at promoting physical activity and healthy nutrition. In line with the duration defined by the complete intervention, the TBHC must last for 18 months. It consists of 13 sessions, each with a duration of 20 to 30 min.

## 2. Theoretical and Empirical Basis

In general, an intervention can be segmented into two parts: (1) activities performed with the target persons, i.e., the core of the intervention, and (2) activities to ensure that the core is realized according to the protocol of the intervention, i.e., measures to ensure the quality of the intervention. In this article, the theoretical derivation focuses on the core of the intervention. The core of this TBHC consists of two parts: (1) the way in which coaches and patients interact with each other and (2) the contents discussed within this interaction. Both parts are developed based on different theories.

### 2.1. Style of Interaction between Coaches and Patients

The recommendations regarding the interaction between coaches and patients are based on (1) the self-determination theory (SDT) of Ryan and Deci [25,26] and (2) motivational interviewing (MI), as developed by Miller and Rollnick [27]. SDT constitutes a theoretical foundation for deriving implications regarding the style of these interactions, while MI constitutes an arsenal of techniques for acting upon these implications. SDT was selected for this purpose because SDT outlines what generally has to be taken into account in interactions with patients to achieve long-lasting behavioral changes [28]. Some MI techniques are used for concretizing the implications of SDT. The reason for this choice is that SDT provides explanations regarding the effects of MI techniques [29] and that, therefore, MI techniques can be seen as possible realizations of the principles implicit in SDT. Adopting MI techniques in this way does not mean adopting the whole approach of MI. Only those techniques that seem appropriate from the perspective of SDT are chosen. 

#### 2.1.1. SDT

SDT was developed by Deci and Ryan [25,26]. They assumed that human behavior is determined by three basic needs: autonomy, competence, and relatedness. The need for autonomy is the need to feel that one is the “acting person” and to behave according to one’s own interests and values. The need for competence encompasses the need to feel effective in any interaction with the social environment and to be able to express and develop one’s own abilities. The need for relatedness is the need to feel accepted and cared for in the social environment, as well as to feel connected to others [25]. According to Deci and Ryan [30], satisfaction of the needs for competence, autonomy, and relatedness leads to a sense of integrity and well-being. Moreover, the more someone feels that a specific behavior serves these needs, the more self-determined that behavior will be.

**Figure 1:**
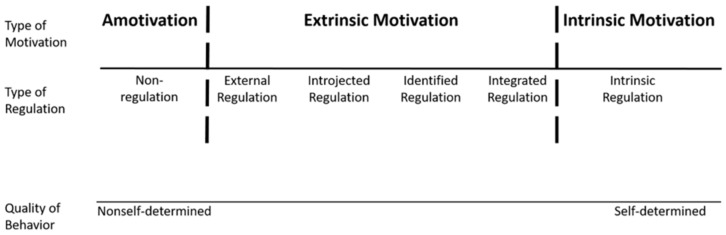
The self-determination continuum, along with the types of motivation and types of regulation. Ryan and Deci [26]; copyright 2002 by Rochester Press.

Deci and Ryan also present a category system for distinguishing the different levels of self-determination. With regard to a specific behavior, they distinguish three types of motivation: amotivation, extrinsic motivation, and intrinsic motivation. In the state of amotivation, a person has no intention of performing the behavior. In the state of extrinsic motivation, a person performs the behavior in order to attain a goal that occurs as a result or consequence of the behavior. In the state of intrinsic motivation, a person performs the behavior because that person likes the behavior itself. The degree of self-determination is lowest for amotivation and highest for intrinsic motivation. Deci and Ryan also distinguish four types of regulation associated with extrinsic motivation: external regulation, introjected regulation, identified regulation, and integrated regulation. External regulation means that the behavior is directly controlled by external stimuli, such as rewards, punishment, or social pressure. Introjected regulation means that the behavior is controlled by some internal regulations that are not yet truly accepted by the acting person. Identified regulation means that the behavior is controlled by values that indicate the personal importance of the behavior. Integrated regulation means that the behavior is controlled by its being in congruence with the self. The degree of self-determination is lowest in external regulation and then increases through introjected and identified regulation to integrated regulation, which has the highest degree of self-determination (Figure 1). As Deci and Ryan explain, ”The more fully a regulation (or the value underlying it) is internalized, the more it becomes part of the integrated self and the more it is the basis for self-determined behavior” [26] (p. 15).

SDT implies that the more self-determined a specific behavior is, the more people will adhere to it. This idea is corroborated by empirical results. People with more self-determined regulations (integrated, identified, intrinsic) show greater adherence to medication regimes [31], better glucose control [32], and more internalized motivation for physical exercise [28]. Moreover, recent meta-analyses indicate that interventions based on SDT improve physical activity behavior and healthy nutrition [33,34].

#### 2.1.2. MI

SDT implies that the coach should address the patient’s needs for autonomy, competence, and relatedness during the coaching procedure. To ensure that this occurs, techniques regarding MI [27] are applied. MI adopts Rogers’ client-centered therapy approach [35] and can be defined as a collaborative, person-centered guiding style of communication for enhancing intrinsic motivation and commitment to behavior change [27]. Miller and Rollnick [27] define four general principles of motivational interviewing: (1) expressing empathy, i.e., building a compassionate, collaborative working alliance centered on the patients’ needs; (2) developing a discrepancy between the patients’ values, their goals, and the current problematic behavior; (3) rolling with resistance, i.e., avoiding coercion and emphasizing the patients’ autonomy; (4) promoting self-efficacy by increasing patients’ belief and confidence in their own abilities and resources regarding change. Miller and Rollnick also present techniques for realizing these four general principles. According to Markland et al. [29], many of these techniques address one of the basic needs postulated in SDT. Regarding the need for autonomy, these techniques are rolling with resistance, avoiding coercion, exploring healthy options, encouraging change discussion, and letting the patients make their own decisions. Regarding the need for competence, these techniques comprise presenting neutral information about behaviors and their outcomes, helping patients to develop realistic goals, providing positive feedback, and supporting self-efficacy. Regarding the need for relatedness, these techniques comprise expressing empathy, exploring the patient’s concerns, demonstrating an understanding of the patient’s position, and avoiding judgment or blame.

MI has been widely used in behavior change interventions for individuals with chronic conditions such as T2DM and CHD. According to several reviews [36,37,38,39,40], these interventions either had no statistically significant effect or had a statistically significant positive effect.

### 2.2. The Contents of the Interaction

The selection of the contents of the coach–patient interaction is based on (1) the health action process approach (HAPA) developed by Schwarzer [41] and (2) the taxonomy of behavior change techniques (BCTs) developed by Michie et al. [42]. Analogously to the preceding sub-chapter, HAPA constitutes the theoretical foundation from which implications for the intervention are derived, while the taxonomy of BCTs constitutes an arsenal from which measures to concretize these implications can be chosen. HAPA has been chosen as the theoretical foundation because it specifies which kind of measures are the most promising for establishing a specific behavior, considering the person’s internal status with regard to this behavior. It has already been applied successfully to persons with chronic conditions [43]. The BCTs have been chosen for concretizing the implications of HAPA because many of the BCTs have already been shown to be effective [44] and because many of these BCTs obviously relate to the concepts applied in HAPA [45].

#### 2.2.1. HAPA

**Figure 2:**
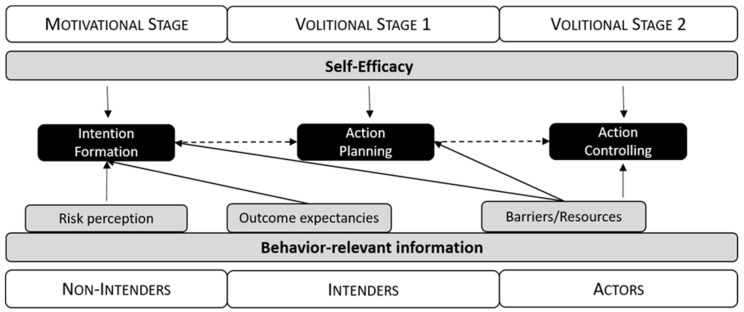
Behavior-relevant information regarding the health action process approach.

HAPA is a social-cognitive behavior change model that addresses the processes of intention formation and of transforming intention into action [41,43]. According to HAPA, this process can be divided into two stages: (1) a pre-intentional motivational stage that addresses the formation of a behavioral intention; (2) a post-intentional volitional stage that addresses the translation of behavioral intention into behavior [46]. The post-intentional stage is further divided into two sub-stages: (1) the consolidation of the intention and preparation of the corresponding action, i.e., action planning; (2) the actual initiation and maintenance of action, i.e., action control. These three stages, i.e., the motivational and the two volitional stages, represent three groups of individuals: (1) non-intenders, i.e., individuals in the motivational stage; (2) intenders, i.e., individuals in the stage of action planning; (3) actors, i.e., individuals in the stage of action control (Figure 2).

The definitions given in HAPA for the different groups imply that the individuals of these groups process behavior-relevant information in a different manner [43]:

- Non-intenders are especially affected by information about their health risks and about the risk-related outcomes of their current and any possible alternative behavior. They balance the positive and negative consequences of both kinds of behavior. The more the balance is in favor of the alternative behavior, the more likely non-intenders are to form an intention to perform that alternative behavior.
- Intenders concretize this intention into an internalized goal and try to develop concrete plans as to how and when to perform the intended behavior. The more they internalize their goal and succeed in developing plans of action, the more likely they are to initiate the behavior.
- Actors are especially susceptible to information regarding barriers that might impede and resources that might facilitate the behavior (e.g., daily routines). The fewer barriers and the more resources they see, the more likely they are to actually maintain the behavior or resume it after a relapse.

Moreover, HAPA also contains central ideas from the social cognitive theory (SCT) of Bandura [47,48]. To be specific, according to HAPA, self-efficacy is a further determinant of intention formation and the performance of behavior [43]. Just as in SCT, self-efficacy is understood to be the person’s belief in being capable of performing a certain kind of behavior; persons are assumed to be more likely to perform a specific behavior if they feel more capable of performing it. Several empirical findings corroborate the assumptions of HAPA [49]. Moreover, interventions that are tailored to the specific needs of individuals belonging to different HAPA groups have been found to be effective for increasing physical activity [50,51] and improving healthy nutrition [52].

#### 2.2.2. BCTs

HAPA presents general recommendations for designing interventions that address behavior change. To concretize these recommendations, the standardized taxonomy of the BCTs developed by Michie and colleagues [42] was applied. BCTs are understood as the “active ingredients” of an intervention that are “observable, replicable, and irreducible” and are “designed to alter or redirect causal processes that regulate behavior” [42] (p. 82). To provide a conceptual system for describing behavior change interventions in a uniform manner, Michie et al. [42] developed a taxonomy of such BCTs via a Delphi survey designed with behavior change experts from multiple disciplines, including psychology, behavioral medicine, and health promotion. The resulting taxonomy presents the most prevalent and effective 93 BCTs, categorized into 16 distinct categories, and includes detailed information for the operationalization of each BCT. The taxonomy of BCTs has already been applied in different settings to identify those BCTs that are especially effective [53,54,55].

## 3. Derivation of the Intervention

In this section, the core of the intervention is derived from the theoretical and empirical basis described above. Additionally, measures to ensure the quality of the intervention are presented. 

### 3.1. Core of the Intervention

According to SDT, the extent to which possible positive effects on physical activity and healthy nutrition continue will depend on how self-determined the regulation is regarding these two kinds of behavior. To increase the level of self-determination, the frequency of the coaching sessions should gradually decrease toward the end of the intervention. This is in line with common practice in most behavior change interventions [56]. Accordingly, in the TBHC presented herein, individual sessions are held every two weeks for the first two months, every four weeks from the third month to the fifth month, and every eight weeks from the sixth month to the eighteenth month of the intervention. According to HAPA, individuals at different stages should be treated differently. Consequently, the session contents are not the same for all patients. Instead, there is a set of coaching tools containing recommendations for conducting a session that addresses a specific HAPA group. From this particular set of tools, the tool can be chosen that seems most appropriate to the present situation of the patient. The decision as to which tool seems most appropriate is made at the beginning of each session, according to an a priori specified structured selection procedure. Both the coaching tools and the selection procedure are designed in such a way that the demands derived from SDT are satisfied. The following sections address first the set of coaching tools and then the procedure for selecting a coaching tool for an individual session. 

#### 3.1.1. Coaching Tools

**Figure 3:**
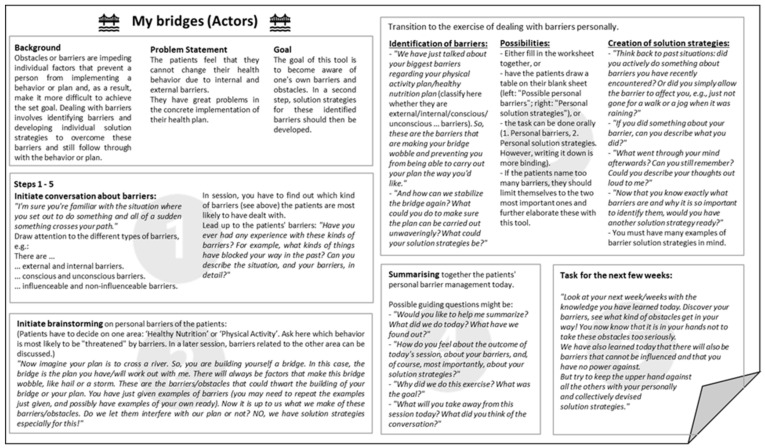
One example of a theory-based coaching tool.

In line with HAPA, coaching tools for four different problem categories are provided. These four categories are: (1) the problems usually encountered by non-intenders; (2) the problems usually encountered by intenders; (3) the problems usually encountered by actors; (4) the problem of lacking self-efficacy. As there are different problems within each of these four categories, four different coaching tools are provided for each category. This results in 16 different tools. The concrete measures applied to solve the problems addressed by the 16 tools have been produced by selecting those BCTs [42] that the developers of the intervention considered to be most effective for the respective purposes (Table 1). In all these coaching tools, one or more than one of the MI techniques that address the needs of SDT (Section 2.1.2) are applied. All coaching tools have the same structure. They begin with (1) background information regarding the problem addressed by the tool, (2) the definition of this problem, and (3) the formulation of a goal that is addressed by the tool. Subsequently, the individual steps that the coach should perform together with the patient during the session are described (see Figure 3 for an example).

#### 3.1.2. Selection of the Coaching Tools for the Individual Sessions

In all but the last two sessions, the coaching tool is selected based on information collected at the start of the session. This information refers to two different issues: (1) current problems with attaining the behavior change goals; (2) the HAPA group assignment. The current problems encompass, on the one hand, general problems with self-efficacy regarding physical activity or healthy nutrition and, on the other hand, specific problems in performing one or both of these two kinds of behavior. The HAPA group assignment is determined separately with regard to physical activity and healthy nutrition, using a modified version of an assessment tool developed by Lippke et al. [50]. In both cases, two questions are formulated. The first question regarding physical activity is: “Are you currently physically active one or more times per week for 20 min or more, for example, walking, gardening, or sporting activity?”. If the answer is “Yes”, the person is classified as an actor. If the answer is “No”, the person is then asked, ”Do you have the firm intention of being physically active one or more times per week for at least 20 min in the next few weeks?” If the answer is ”Yes”, the person is classified as an intender; otherwise, they are classified as a non-intender [50]. With regard to healthy nutrition, the consumption of two servings of fruits and vegetables one or more days per week is substituted for 20 min of physical activity. 

**Figure 4:**
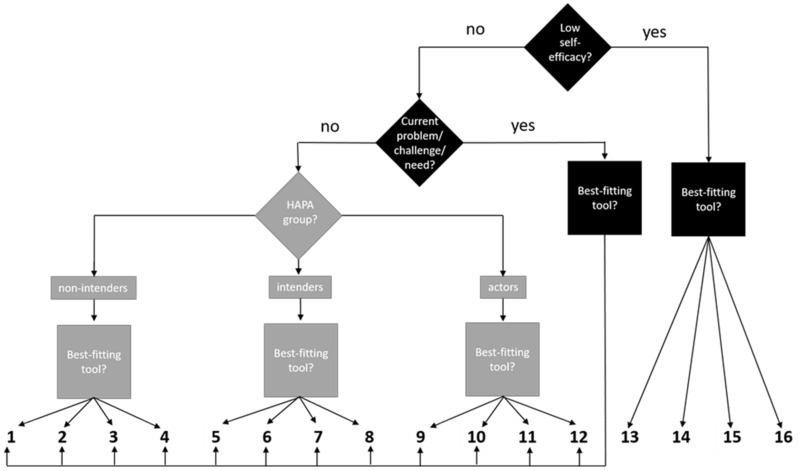
Decision tree for the selection of a coaching tool.

If the patient has problems with self-efficacy, a coaching tool referring to self-efficacy is selected (Figure 4). If the patient has a specific problem regarding the health behavior change, the most appropriate of the coaching tools addressing behavior-relevant information (Table 1) is selected. If the patient has no current problems to report, the coach and patient select a coaching tool according to the patient’s HAPA group assignment (Figure 4). If the patient’s HAPA group assignment differs for physical activity and healthy nutrition, the behavior with the HAPA group assignment that is farthest away from “actor” is addressed, and a coaching tool matching the HAPA group assignment for this behavior is selected. If the HAPA group assignment is the same for both behaviors, the coach and patient will decide, mainly based on the patient’s preferences, which behavior is to be discussed. The coach then selects a tool belonging to that HAPA group. As far as possible, the patient’s preferences are taken into consideration in all decisions made during the selection process, as is directly implied in both SDT and MI. The coaching tools “Self-reflection” and “My future self-management” are mandatory for the last two sessions of coaching. From the perspective of SDT, these two tools serve to promote the patient’s internalization of the regulations with regard to physical activity and healthy nutrition and, thereby, increase their self-determination. From the perspective of HAPA, both tools serve to support action control. Both should also help the patients to continue with physical activity and healthy nutrition in the period after the coaching has finished.

### 3.2. Measures to Ensure Quality

To ensure the quality of the TBHC, (1) coaches are prepared in an intensive training program prior to TBHC, and (2) regular supervision sessions are performed during TBHC. The training prior to the interventions takes 40 h. The contents of this training encompass information on the background of the program, along with support for understanding and applying the theories, as well as understanding and applying the coaching tools. Moreover, coaches practice the coaching strategies used in role-plays with trained simulation patients. Evaluation of the training is described in detail elsewhere [57].

The supervision sessions are conducted with different personnel at weekly intervals, at monthly intervals, and, additionally, on-demand for critical cases. The weekly sessions are performed by the psychosomatic core group, which consists of the coaches (trained sports or nutritional scientists), a psychologist, and a senior physician who is a specialist in psychosomatic medicine, internal medicine, and communication training. In these sessions, coaching cases are presented and discussed. The monthly supervision sessions are performed by the plenary, i.e., the psychosomatic core group along with the health behavior scientist who has developed the coaching tools for the TBHC. In these sessions, coaching cases are discussed extensively regarding appropriate tool selection. In cases in which the basic needs are not sufficiently satisfied, the plenary discusses which MI techniques or communication skills can be used to ensure the satisfaction of these basic needs, as well as to increase self-determination. The critical cases are discussed regarding their current challenges, e.g., tool selection, MI techniques, and needs support. In summary, the supervisions are in place to verify and guarantee fidelity to the protocol of the entire TBHC, including all combined techniques and methods.

## 4. Discussion

The main objective of this article was to attempt a new format to allow for a detailed description of an intervention and, especially, its theoretical derivation. A manuscript laid out according to this format consists of (1) an introduction, (2) a presentation of the theoretical and empirical basis of the intervention, (3) a detailed description of the intervention, including its theoretical derivation, and (4) a discussion. Using this format, it was possible to elaborate upon how the intervention was derived from theories. The derivation is based on a very general conception of an intervention. According to this conception, an intervention consists of (1) its core and (2) measures to ensure its quality. The core of a TBHC, in turn, consists of (1) the way in which coaches and patients interact with each other and (2) the contents discussed within this interaction. Conceptualizing the core in this way is especially crucial for the derivation of the intervention. Conceptualizing it in a different way might lead to a selection of different theories and different empirical findings as fundaments of the intervention. This, in turn, might lead to the development of a very different intervention. Whether the conception applied here is sufficient or whether it should be modified and, if yes, how it should be modified might be a topic of further theoretical discussion regarding intervention development.

A further decision that has determined this intervention is the choice of the theories applied as a theoretical fundament. These comprise the use of SDT as the theoretical fundament for substantiating the style of interactions between coaches and patients and the use of HAPA as the theoretical fundament for substantiating the contents discussed within these interactions. SDT has been chosen because it describes the factors that determine how much an individual internalizes a specific behavior and because these factors can be operationalized via the way in which the coaches interact with the patients. Moreover, SDT is presently the most widely accepted theory that serves this purpose. HAPA has been chosen because it specifies which contents are important for the patients, depending on their present relationship to the behavior in question. In contrast to SDT, HAPA is not the most widely accepted theory that serves the purpose for which it has been chosen. An alternative theory serving the same purpose is the transtheoretical model (TTM) [58]. However, HAPA has been chosen in preference to the TTM because the assumptions regarding the cognitive processes involved are more fully elaborated upon in HAPA than in the TTM. What theories should best be applied in this context might be a further topic for theoretical discussion regarding intervention development.

The TBHC presented here is currently evaluated as part of the intervention in which it is included. As mentioned above, the complete intervention consists of peer-support group meetings, personalized patient feedback, access to a web portal, and the TBHC. Moreover, the TBHC itself is only administered to a sub-sample of the persons addressed by the complete intervention, i.e., to those persons with low health competency and/or low activation level. This complete intervention is evaluated using a randomized controlled trial (RCT), with one study group receiving the intervention and the other receiving regular care [21]. The results of this trial inform researchers about the effectiveness of the complete intervention, but not about the effectiveness of its individual parts. In other words, if the complete intervention is effective, this might be partly due to the effectiveness of the TBHC. Evaluating the effectiveness of the four components of the complete intervention would require a study design comprising four factors with two levels, i.e., an RCT with 16 study groups. Such an RCT would be completely infeasible. This problem applies to virtually all complex interventions.

## 5. Conclusions

Further development of these interventions may benefit from an explicit discussion of their theoretical fundaments. The meta-conceptions applied here, i.e., the structure of the manuscript and the general conception of an intervention, can be applied to structure further theoretical derivations. The theoretical derivation presented in this article can be taken as an example of how this task can be accomplished.

## Figures and Tables

**Table 1:** Coaching tools, their goals, and the underlying behavior change techniques.

	No.	Coaching Tool Name and Goal	Behavior Change Technique (BCT)
**Coaching Tools** **for** **Non-intender**	1	Activity check Patients increase their awareness of their current level of physical activity, in terms of risk perception. Patients form an intention to become more active and, thus, undertake more for their own health.	Feedback on behavior
	2	My activity type Patients create an activity profile in order to typify, summarize, and, subsequently, reflect on their own ideas about physical activity.	Instruction on how to perform the behavior
	3	Decision balance Patients identify and increase their awareness of both the positive consequences of health behavior change and the negative consequences of continuing with their current behavior.	Pros and cons
	4	My health motivation Patients identify their own level of self-determination in terms of physical activity/healthy nutrition and develop ways in which they can strive for a more autonomous form of regulation.	Feedback on behavior
**Coaching Tools** **for** **Intender**	5	My health plan Patients translate their goals into precise action plans by independently concretizing the “what, when, where, and, possibly, with whom?” of their health plan.	Action planning
	6	My health diary Patients document their physical activity/healthy nutrition and the positive consequences and feelings during and after; they are, thus, made aware of the connection between greater physical activity/healthier nutrition and their mood.	Self-monitoring of behavior
	7	My health goal Patients use the SMART method tool to set a clear, unambiguous, and self-concordant goal.	Goal setting
	8	Visualization of my health goal Patients use visualization techniques to help themselves to further internalize their previously defined goal by “imagining” the achievement of their goal.	Goal setting
**Coaching Tools** **for** **Actors**	9	My bridges Patients become aware of their own internal and external barriers and develop appropriate solution strategies for the barriers they identify.	Problem-solving
	10	My health routine Patients develop a routine in their desired area (healthy nutrition/physical activity) that fits their wants/needs/everyday life and that they can integrate long-term into their daily routine.	Habit formation
	11	Self-reflection (scheduled for the 11th coaching session) Patients revisit and reflect on the greatest challenge to their behavior change that they have faced so far during the coaching process, to prepare for everyday life without the coach.	Focus on past success
	12	My future self-management (scheduled for the 12th coaching session) Patients review and consolidate the proven coaching tools and practical techniques they have learned and developed for their own health behavior change, to underline the importance of future planning in preparation for everyday life without the coach.	Action planning

## Study Preregistration Information

Trial registration number of the whole pragmatic randomized controlled trial (in which the TBHC is embedded): 00020592.
